# Regression of oesophageal varices in total anomalous pulmonary venous connection

**DOI:** 10.1093/icvts/ivab317

**Published:** 2021-11-19

**Authors:** Shingo Takahara, Naoki Masaki, Hideki Tatewaki, Sadahiro Sai

**Affiliations:** Department of Cardiovascular Surgery, Miyagi Children’s Hospital, Sendai, Miyagi, Japan

**Keywords:** Total anomalous pulmonary venous connection, Pulmonary vein obstruction, Oesophageal varices

## Abstract

The coexisting of oesophageal varices with total anomalous pulmonary venous connection is extremely rare but contains a potential leading to a lethal haemorrhage. The fate of the oesophageal varices after total anomalous pulmonary vein connection repair remains largely unknown. We herein report a case with infracardiac type total anomalous pulmonary venous connection with remarkable oesophageal varices. In the present case, of note, the oesophageal varices were completely regressed after total anomalous pulmonary venous connection repair without any intervention. This case might help a surgical team reduce the hesitation to repair the total anomalous pulmonary venous connection regardless of oesophageal varices, a potentially fatal condition.

## INTRODUCTION

Pulmonary venous obstruction (PVO) in total anomalous pulmonary venous connection (TAPVC) is a life-threatening complication, which causes congestion of pulmonary veins and consequent post-capillary pulmonary hypertension. While PVO occurs most frequently in infracardiac type of TAPVC, the mechanisms and sites of PVO may vary from a case to a case and so do the consequences of PVO.

Oesophageal varices associated with TAPVC are extremely rare [1], but it contains a risk of life-threatening haemorrhage [2]. However, the fate of oesophageal varices after TAPVC repair remains unclear.

We herein report a case of the oesophageal varices with infracardiac type TAPVC, which showed the complete regression after TAPVC repair.

## CASE REPORT

**Figure 1:**
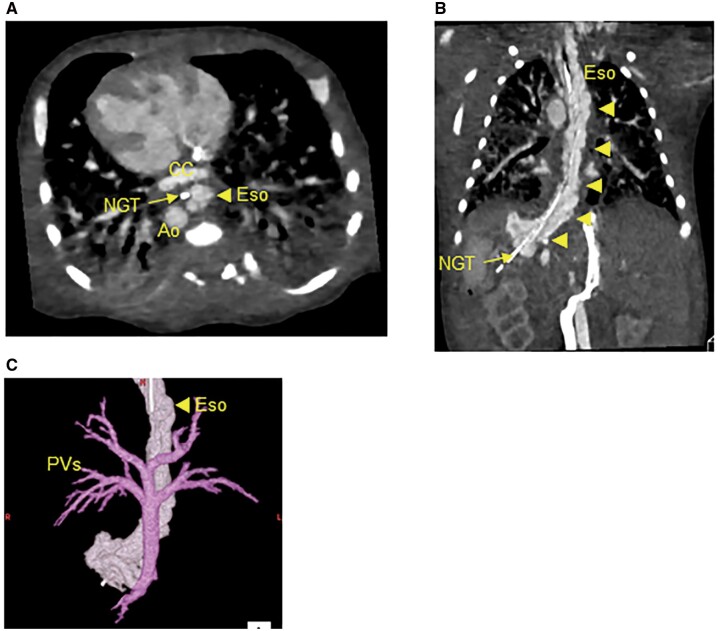
Remarkably dilated oesophageal varices in preoperative computed tomography. (**A**) The axial image, (**B**) the coronal image and (**C**) the constructed 3D image of preoperative computed tomography. Arrowheads indicate the oesophageal varices. Ao: aorta; CC: common chamber; Eso: oesophagus; NGT: nasogastric tube; PV: pulmonary vein.

**Figure 2:**
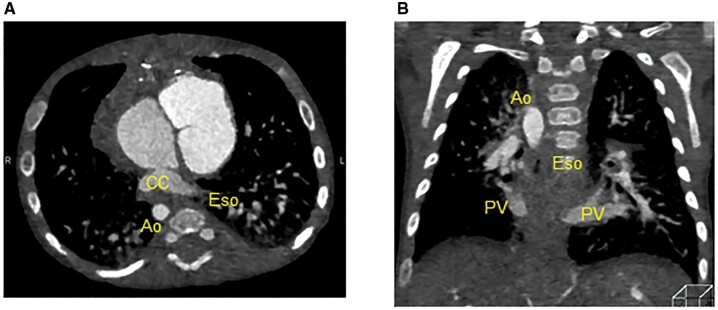
The complete regression of the oesophageal varices in postoperative computed tomography. (**A**) The axial image, (**B**) the coronal image of postoperative computed tomography. Ao: aorta; CC: common chamber; Eso: oesophagus; PV: pulmonary vein.

A male infant born at 36 weeks of gestation and weighing 2.6 kg was diagnosed prenatally with heterotaxy syndrome, complete atrioventricular septal defect and infracardiac type TAPVC. The echocardiography after birth revealed severe PVO at the vertical vein draining into the portal vein. The enhanced computed tomography demonstrated that the patient had the extensively dilated oesophageal varices (Fig. 1A–C) without any evidence indicating hepatic abnormality nor portal vein obstruction that can cause oesophageal varices. Although his vital was stable, since the PVO was severe, he underwent the TAPVC repair at the following day of birth despite the oesophageal varices contained a risk of lethal haemorrhage under systemic heparinization. The conventional TAPVC repair with anastomosis of the left atrium and common chamber along with ligation of the vertical vein was performed under the standard cardiac arrest with moderate hypothermia. Since prolonged surgery under full heparinization was deemed to be high risk as well as his body size was small, pulmonary artery was tightened with body weight + 15 mm in circumference length. At the following day of the surgery, the progressive hypoxaemia mainly due to temporary respiratory dysfunction by perioperative pulmonary congestion and surgical insult required extracorporeal membrane oxygenation support. Because of not only oesophageal varices but also the haemorrhage tendency, unfractionated heparin infusion was carefully commenced and increased up to 20 units/kg/h to maintain 110–120 s in active coagulation time. As the result, the patient was successfully separated from it 3 days after the installation with no gastro-oesophageal haemorrhage. The postoperative echocardiography confirmed unrestrictive pulmonary vein flow at the anastomosis site of the common chamber and the left atrium. Of note, the postoperative computed tomography demonstrated that the complete regression of the oesophageal varices (Fig. 2A and B) without any interventions.

## DISCUSSION

The oesophageal varices with TAPVC are extremely rare [1]. Moreover, the fate of oesophageal varices has not been well described in previous reports [2–4]. The present case demonstrated for the first time that the oesophageal varices might be regressed by decompression of venous pressure around gastric veins, which are likely a source of blood flow to varices, with repair of TAPVC.

Since the portal vein is the most common site where the anomalous vertical vein in infracardiac type of TAPVC is drained, portal venous hypertension could happen due to an obstruction after margining site of the vertical vein and the portal vein as well as excessive blood flow to the portal vein [3]. When portal venous hypertension occurs by either mechanism, all vessels on upstream of the culprit site can be congested, which might cause oesophageal varices [5].

In the present case, the varices appear to be developed by excessive blood flow through the portal vein, since the varices were completely regressed after TAPVC repair, which reduces the blood flow to the gastro-oesophageal venous junction through portal vein. The present case might help a surgical team overcome hesitation to perform TAPVC repair in a case with the oesophageal varices with the expectation of their regression.

**Conflict of interest:** none declared.

**Funding:** none. 

## Reviewer information

Interactive CardioVascular and Thoracic Surgery thanks the anonymous reviewers for their contribution to the peer review process of this article.
